# Feasibility of Ecological Momentary Assessment Using Cellular Telephones in Methamphetamine Dependent Subjects

**DOI:** 10.4137/sart.s428

**Published:** 2008-02-10

**Authors:** Gantt P Galloway, Ryne Didier, Kathleen Garrison, John Mendelson

**Affiliations:** Addiction Pharmacology Research Laboratory, California Pacific Medical Center, San Francisco, CA 94110, U.S.A.

## Abstract

**Background:**

Predictors of relapse to methamphetamine use are poorly understood. State variables may play an important role in relapse, but they have been difficult to measure at frequent intervals in outpatients.

**Methods:**

We conducted a feasibility study of the use of cellular telephones to collect state variable data from outpatients. Six subjects in treatment for methamphetamine dependence were called three times per weekday for approximately seven weeks. Seven questionnaires were administered that assessed craving, stress, affect and current type of location and social environment.

**Results:**

395/606 (65%) of calls attempted were completed. The mean time to complete each call was 4.9 (s.d. 1.8) minutes and the mean time to complete each item was 8.4 (s.d. 4.8) seconds. Subjects rated the acceptability of the procedures as good. All six cellular phones and battery chargers were returned undamaged.

**Conclusion:**

Cellular telephones are a feasible method for collecting state data from methamphetamine dependent outpatients.

## Introduction

Relapse to drug use is a dynamic and fluctuating process in which factors associated with use may be forgotten or inaccurately recalled if collected retrospectively. Methods that assess risk factors and drug use at frequent intervals may permit better characterization of the contribution of risk factors to relapse compared to retrospective self-reports obtained on less frequent occasions. Until recently, efforts to collect frequent measures have been impeded by two issues: the inability to assess drug users in their natural environment at times proximal to the relapse event and the lack of statistical methods to analyze repeated-measures data in which missing data points are common. Theoretically, obtaining measures of state variables close to the time they occur should be possible. Cellular telephones are a widely available technology that could permit frequent measurement of state variables. This type of recurrent, in situ assessment, known as Ecological Momentary Assessment (EMA) or Experience Sampling, allows real-time assessment of environments and experiences of research subjects. EMA can eliminate recall bias, enable a more finely tuned focus on the real-time antecedents of each episode of drug use and can be tested in a repeated measures model, considerably increasing statistical power. Although EMA has been demonstrated to be a valuable method of collecting data from subjects in a range of contexts, it has not yet been tested with methamphetamine (MA) users.

Previous studies of relapse to drug use have utilized a variety of methods to collect EMA. For example, programmable wristwatches were used to collect EMA with 27 alcohol-dependent outpatients in a 3-week study. Unfortunately, subjects reported poor adherence; even those who accurately recorded the date and time of the eight daily prompts nonetheless often completed the survey questions at later times [1]. In part because the design included subject-initiated calls, the overall rate of adherence cannot be calculated. Shiffman et al. implemented the use of electronic hand-held computerized devices to collect EMA from 304 smokers enrolled in a smoking cessation clinic [2, 3]. Participants were prompted randomly during subject-specific waking hours for 16 and 14 days, respectively. Although subjects responded within two minutes 91% of the time, data were only collected for 7.9 ± 0.4 days. Insufficient data were presented to calculate how many calls were planned and thus the rate of adherence cannot be calculated. The use of electronic hand-held computerized devices was implemented to collect EMA from 18 alcohol-dependent subjects hospitalized, full-time or part-time, for detoxification [4]. Random prompts were programmed to fall outside each subject-specific sleep schedule for three weeks. Based upon the number of hours the subject was awake, eight to twelve assessments per day were scheduled for three weeks, with an average of 16.2 ± 1.7 days. Four subjects were excluded due to lost or damaged equipment. The percentage of missing data by subject ranged from 5.6 to 26.8% during the period of participation. Of the approximately 3,780 planned calls, 1,767 (47%) were completed.

Collection of EMA using computerized interactive voice response (IVR) with cellular telephones was compared to paper-and-pencil self-monitoring with 20 social drinkers during a two-week period. While both methods produced similar adherence rates, confidence in IVR data was higher due to the ability to verify data entry date and time [5]. Because the design included subject-initiated calls, the overall rate of adherence cannot be calculated; however, of 560 planned calls to subjects at random times, 517 (92%) were completed. A similar and more recent study [6] assessed the feasibility of utilizing cellular telephones to collect EMA from cocaine-addicted homeless subjects during a two-week intensive outpatient treatment program using automated computerized surveys conducted by IVR technology. Of the 3,360 planned calls, 2,191 (65%) were completed. Although the calls were made throughout the day and night, resulting in high response burden, the good completion rate points to the practicality of utilizing this method with drug-dependent populations. Ultimately, EMA offers unique advantages over retrospective reports including ecological validity, avoidance of recall bias, prevention of pseudo-adherence and flexibility in data collection.

This pilot study was designed to assess the feasibility of the use of cellular telephones in capturing EMA from MA users and, to our knowledge, is the first to employ these methods with MA dependent subjects. Areas of interest included 1) adherence to the protocol, 2) time required to complete the assessments and 3) subject acceptability of the procedure.

## Methods

### Subjects

Six MA dependent outpatients were recruited from a larger study in which a similar battery of assessments was administered on a weekly basis. They were screened for inclusion criteria, including reported MA use within the past 30 days, diagnosis of MA dependence via DSM-IV criteria, and a minimum age of 18 years. All subjects submitted at least one MA-positive urine sample during screening. Subjects were excluded if they were currently DSM-IV-dependent on any other psychoactive substance (except nicotine); had a lifetime history of schizophrenia, schizophreniform, or schizoaffective disorder; or had severe major depression, severe posttraumatic stress disorder, mania, or hypomania within the prior 90 days. In addition, subjects were excluded if they reported more than four hours per week of commitments that would prevent them from answering the phone. Clinical characteristics of participating subjects are presented in Table 1.

### Measures

Seven questionnaires assessed craving, stress, affect, and current type of location and social environment of the subject. These questionnaires were divided into four sets (Set A: Depression Anxiety and Stress Scales [7, 8] and Perceived Stress Scale [9]; Set B: Environmental Circumstances Scale (modified from an unpublished scale provided by K.L. Preston) and Desires for Speed Questionnaire [10]; Set C: Hassles Scale [11]; Set D: Amphetamine Withdrawal Questionnaire [12, 13] and Positive and Negative Affect Schedule [14]. The time period addressed was either the time since the last scheduled assessment (Hassles Scale and Environmental Circumstances Scale) or current state (now) for all other questionnaires. Each time a subject was asked to complete a set of rating scales by cellular telephone, they were also asked if they had used MA since the last scheduled assessment and, if so, how much. Upon completion of the study, subjects were asked to complete a 20-item satisfaction survey.

### Procedure

Subjects were scheduled to receive three calls each weekday for approximately seven weeks. In addition, subjects were offered nine Motivational Enhancement Therapy sessions. Cellular telephones and battery chargers were provided for the subjects with password-protected key locks preventing any outgoing calls except to our laboratory and 911. Subjects were called semi-randomly during each of three 160-minute periods between 10 am and 6 pm, with at least 60 minutes between each call. Calls to subjects were made by research technicians rather than by an automated system, hence the restriction to weekdays between 10 am and 6 pm. In the event that subjects did not answer the cellular telephones when called, up to five additional calls were made at five minute intervals after the scheduled time; subjects were also instructed to call in to the laboratory to complete rating scales during this period if they missed a call. Subjects were asked to complete a set of approximately one-quarter of the scales at each call, each set including a similar number of items. One sequence of sets was randomly generated with a block size of four; this sequence was assigned to all subjects. Subjects were provided an answer card listing the answer options for each instrument. Subjects received $3 per completed call, $40 for returning the cellular telephone and $10 for returning the charger.

## Results

### Protocol adherence

**Figure 1:**
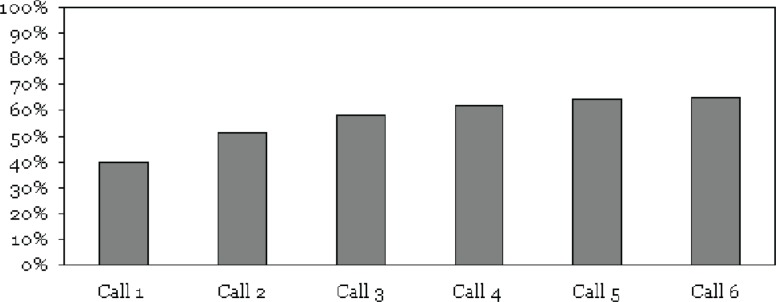
Cumulative call completion.

Subjects completed 395/606 (65%) of the calls assigned when they entered the study (see Fig. 1). 30 calls not made due to technician scheduling conflicts are not included in the 606 calls assigned. Including all scheduled calls yields a total adherence of 395/636 (62%). Of the calls completed, 240/395 (61%) were completed on the first attempt to contact the subject, 373/395 (94%) of the calls were completed by the fourth attempt and 391/395 (99%) were completed by the fifth attempt (see Fig. 1). All six cellular phones and battery chargers were returned undamaged. One subject withdrew from the study after six weeks in order to travel.

### Time required

The mean time to complete a call was 4.9 (s.d. 1.8) minutes. The mean time required per item was 8.4 (s.d. 4.8) seconds over the course of the study. There was no change in the amount of time required to complete calls at the beginning of the study compared to calls at the end of the study.

### Acceptability of study procedures

The five subjects who remained enrolled in the study until the final study visit were asked to complete a satisfaction survey (see Table 2). These subjects reported few problems or inconveniences associated with the phone calls. Subjects reported receipt of proper information regarding the procedures for completing each call. One subject felt “quite a bit” uncomfortable answering personal questions over the phone but the remaining participants felt no discomfort at all. The frequency of the calls was “not at all” bothersome for all subjects except one who reported being “a little bit” bothered, indicating low response burden. All subjects reported no change in ability to answer questions honestly on the phone as opposed to during weekly laboratory visits and claimed to have not provided any inaccurate statements in order to speed up the phone calls. Subjects also reported that they were “quite a bit” more able to remember events when asked about a few hours instead of a whole week. Lastly, being paid $3 per call and $50 for return of the cellular telephone and charger seemed fair to the subjects.

## Discussion

The mean call duration of 4.9 minutes was considered quick by most subjects and other measures of satisfaction with the study were generally high. The ratios of calls completed to calls planned, 62%, and calls completed to calls attempted, 65%, appear to be similar to other EMA studies, although direct comparisons are difficult as some authors appear to have excluded potential calls that were not completed due to subject dropout in their calculations, whereas we did include potential calls that were not completed due to subject dropout in our calculations. In addition, direct comparisons with designs in which subjects initiate calls in response to events such as craving or drug use are problematic because of the difficulty assessing how many such events occurred. It is important that investigators using EMA report adherence clearly and include the number of calls planned. While others have reported problems with loss or damage to personal digital assistants used to collect EMA data [4], all of the cellular telephones we provided to subjects were returned undamaged. We speculate that the monetary incentive offered for return of the telephones played a role in this 100% return rate and recommend use of incentives in future studies. In contrast to a previous study of homeless cocaine users [6], our subjects denied providing any inaccurate statements in order to speed up the calls.

In this study research technicians made the calls during which EMA data were collected. Although the ease of set-up and minimal monetary investment was suitable for a small pilot study, the required technician time would not be practical for larger studies. In larger studies automated data collection methods are likely to be more cost-effective and may have the advantage of yielding more accurate responses [15], as well as facilitating data collection during evening and weekend hours. Methamphetamine use may differ during weekend and evening hours as compared to weekdays between 10 am and 6 pm. Although our staff were not available to make calls during evening and weekend hours, it will be important to implement a system that can collect data during these times. In addition, our requirement that subjects be available for at least 90% of the time during which calls were scheduled limits generalizability. This requirement was imposed because scheduled activities that could interfere with data collection, such a work and school attendance, are likely to occur on weekdays between 10 am and 6 pm. If calls were automated, this requirement could be loosened or eliminated and calls could therefore also be made during evening and weekend hours. Automated data collection may also speed responses by eliminating attempts to engage technicians in conversation.

We made up to 6 attempts at 5-minute intervals to complete each call in an attempt to maximize data collection. However, multiple attempts to collect data raise the issue of the extent to which subjects’ state may have changed in some nonrandom fashion before the call was completed. Comparison of prompt versus delayed responses will provide valuable information on how to implement EMA, but requires a larger data set than that presented here. It does appear that multiple attempts are necessary for reasonable call completion rates but that 1–2 fewer attempts could be made with minimal reduction in rates.

One limitation of this study is the inherent unreliability of cellular telephones. It is likely that our call completion rate is an underestimate of the proportion of calls answered by subjects as not all calls attempted on cellular telephone networks are completed. Data collection programs that run on either personal digital assistants or as programs resident on cellular telephones would eliminate this issue, although those approaches also have drawbacks and the extent of this issue is unclear. A more significant limitation of this study is the small sample size. A larger sample would increase confidence in estimates of the variables examined in this study. A larger sample would also permit evaluate of methodological issues such the optimal number of attempts to complete each call, as noted above, and permit comparison of MA self-report data to conventional measures of MA use such as timeline follow-back [16] and urine toxicology. Despite these limitations, collection of EMA data from MA dependent subjects appears feasible.

## Conclusions

This pilot study demonstrated the feasibility of using cellular telephones to collect EMA data from MA dependent subjects. Protocol adherence was good and the data collected will provide valuable guidance for future studies using cellular phones to collect EMA data. Subjects found 35 items per call acceptable, viewed $3 compensation per call as fair and multiple attempts to complete each call resulted in markedly higher data collection rates. EMA holds promise for investigating the relationship between state variables and use of MA.

## Figures and Tables

**Table 1:** Clinical characteristics.

**Age**	
Median (range)	40 (32–45)
**Gender**	
Male	4 (66.7%)
Female	2 (33.3%)
**Sexual orientation**	
Hetrosexual	4 (66.7%)
Bisexual	1 (16.7%)
Homosexual	1 (16.7%)
**Race**	
Caucasian	6 (100%)
**Usual route of administration**	
Injection	2 (33.3%)
Inhalation	3 (50%)
Insufflation	1 (16.7%)
**Days of MA use in the past 30 days**	
Median (range)	16 (2–28)

**Table 2:** Subject satisfaction survey.

	Mean (s.d.)	Median (range)
1. The cell phone was easy to use.	3.6 (1.3)	3 (2–5)
2. I was given proper instructions on how to operate the cell phone.	4.4 (0.9)	5 (3–5)
3. I had problems with the cell phone.	2.6 (1.1)	3 (1–4)
4. It was inconvenient to receive three calls a day.	1.2 (0.4)	1 (1–2)
5. I was bothered by the constant calls.	1.4 (0.9)	1 (1–3)
6. Having to talk about my feelings three times a day helped me sort out my emotions.	3 (1.2)	3 (2–5)
7. I was properly informed when given the cell phone on the procedure of this experiment.	4.6 (0.9)	5 (3–5)
8. The cell phone calls were quick.	3.6 (1.5)	4 (1–5)
9. The cell phone was easy to answer.	4.2 (0.8)	4 (3–5)
10. I was more able to remember events when asked about a few hours instead of a whole week.	4 (0.7)	4 (3–5)
11. I understood all of the questions that were being asked of me.	4.2 (0.8)	4 (3–5)
12. I felt uncomfortable answering personal questions over the cell phone.	1.8 (1.3)	1 (1–4)
13. It was easier to be honest when answering questions on the cell phone than when I was in the office.	1 (0.0)	1 (1–4)
14. I would have liked to have had a different ring or different settings on my cell phone.	3.5 (1.7)	4 (1–5)
15. It would have been easier if the calls had been made by an automated voice, and I had answered with keystrokes.*	1.8 (1.3)	1 (1–4)
16. I purposefully did not answer the cell phone when 1 didn’t feel like talking.	2.2 (1.6)	2 (1–5)
17. I answered inaccurately in order to speed up the call.	1 (0.0)	1 (1–1)
18. The compensation I received was accurate.	5 (0.0)	5 (5–5)
19. The compensation for the cell phone calls seemed fair.*	5 (0.0)	5 (5–5)
20. The voice of the interviewer was clear and easy to understand.	4.4 (0.9)	5 (3–5)

* Completed by 4 subjects.

**Scale:** 1-Not at all, 2-Little bit, 3-Somewhat, 4-Quite a bit, 5-Extremely.
